# The Lithuanian multiple sclerosis registry: current framework and quality challenges

**DOI:** 10.3389/fneur.2026.1728596

**Published:** 2026-02-23

**Authors:** Julija Isačenko, Dalia Musneckienė, Rasa Kizlaitienė, Nataša Giedraitienė, Romualdas Kizlaitis, Lina Malcienė, Dalius Jatužis, Gintaras Kaubrys, Daiva Rastenytė

**Affiliations:** 1 Department of Neurology, Lithuanian University of Health Sciences Kaunas Clinics, Kaunas, Lithuania; 2 Department of Neurology, Medical Academy, Lithuanian University of Health Sciences, Kaunas, Lithuania; 3 Clinic of Neurology and Neurosurgery, Institute of Clinical Medicine, Faculty of Medicine, Vilnius University, Vilnius, Lithuania; 4 Independent Researcher, Vilnius, Lithuania; 5 Department of Neural Diseases and Rehabilitation, Klaipeda University Hospital, Klaipeda, Lithuania

**Keywords:** epidemiology, Lithuania, multiple sclerosis, real-world data, registries

## Abstract

**Objectives:**

This article presents the current structure, data collection methods, coverage, and limitations of the Lithuanian Multiple Sclerosis (MS) Registry.

**Materials and methods:**

The Lithuanian MS Registry, established by the Lithuanian Neurologists' Association, began its activities in 2013. All three national centers providing MS treatment in Lithuania have secure access to their respective patient data and can enter new clinical information into the registry. Adult individuals with a suspected or confirmed diagnosis of MS who receive care in Lithuania and provide written informed consent are eligible for inclusion in the Multiple Sclerosis Patient Surveillance System registry. For the present analysis, a dataset comprising all records from February 1, 2013, to January 1, 2024, was extracted on June 17, 2024.

**Results:**

The registry collects individual data on demographics, results from specific diagnostic procedures (such as cerebrospinal fluid analysis, evoked potentials), clinical evaluations conducted at every visit (based on the Expanded Disability Status Scale), treatment, and relapses (including their dates and whether corticosteroid treatment was administered). Currently, the registry includes 2,923 patients. Of these, 1,651 patients are classified as in active follow-up (those with at least one recorded visit since January 1, 2021) and represent less than half of the total MS population in Lithuania (47.3%). The registry covers approximately 69.5%−74.6% of all patients receiving disease-modifying therapy in the country.

**Conclusion:**

Although the registry has been operating for more than a decade, challenges remain in patient enrollment and ensuring high-quality data collection. Strengthening validation processes is essential to ensure the registry's reliability and utility in both clinical practice and research settings.

## Introduction

Patient registries serve as valuable tools for generating meaningful data that guide healthcare policymakers in developing effective models of care (1). In the rapidly evolving field of multiple sclerosis (MS) treatments, registry-based real-world studies are increasingly important in informing clinical decisions (2–4). These studies often provide insights more applicable to the general population than randomized controlled trials, offering practical information on the effectiveness and safety of disease-modifying therapies (5). Moreover, MS registries facilitate international data sharing, enabling researchers and institutions worldwide to collaborate on studies that enhance the understanding of MS across diverse populations (6–8).

Numerous European countries have national MS registries: Denmark (since 1956), France (since 1976), Sweden (since 1997), Norway (since 1998), Germany (since 2001), Italy (since 2001), Austria (since 2004), United Kingdom (since 2009), Poland (since 2011), Belgium (since 2012), the Czech Republic (since 2013), Switzerland (since 2012), Finland (since 2014), the Netherlands (since 2017), et al. (6, 9–14).

The Lithuanian MS Registry, established by the Lithuanian Neurologists' Association, began its activities in 2013. Although the registry has been operating for more than a decade, it still faces challenges in enrolling patients and obtaining high-quality data.

The purpose of this article is to introduce the Lithuanian MS Registry and to evaluate its current performance by describing its structure and data collection processes, assessing patient coverage and data quality, and identifying limitations affecting its representativeness of the national MS population.

## Materials and methods

The Lithuanian Neurologists' Association initiated and funded the Lithuanian MS Registry—an open-ended, longitudinal, prospective, nationwide, patient-oriented registry study. Data collection for biomedical research was approved by the Lithuanian Bioethics Committee in 2011.

An information system was developed to align with the data structures of the MSBase and iMed platforms, while also accommodating the specific requirements of Lithuanian MS specialists. The registry is built on a Microsoft SQL Server database with secure web-based access and is hosted at the Vilnius University Hospital Data Center.

Adult individuals with a suspected or confirmed diagnosis of MS (based on the 2017 McDonald criteria) who receive care in Lithuania and provide written informed consent are eligible for inclusion in the Lithuanian MS Registry.

All three national centers providing MS treatment in Lithuania—Vilnius University Hospital Santaros Klinikos, the Hospital of the Lithuanian University of Health Sciences Kauno Klinikos, and Klaipeda University Hospital—have secure access to their respective patient data and can enter new clinical information into the registry. Although physicians cannot access data from other centers, the registry is designed to maintain data integrity, prevent duplicate entries by checking personal codes when new patient data are added, and ensure continuity of care during patient transfers between centers or treating physicians. Registry records were completed by a neurologist, resident, and secretary. Data entry was performed both prospectively and retrospectively using medical records, reflecting real-world clinical data and monitoring of patient care. This included all documented patient visits—whether scheduled or conducted remotely—typically occurring every 3 months. Patients could also be seen earlier if they felt it necessary.

The registry collects individual-level data on demographic and socioeconomic characteristics, including age, sex, education level, marital status, and employment status; clinical history, including date of first symptom onset, date of diagnosis, and date of first clinical visit; diagnostic investigations, including cerebrospinal fluid analysis and evoked potentials; clinical assessments performed at each visit using the Expanded Disability Status Scale (EDSS); disease-modifying treatments; and relapse history, including relapse dates and the use of corticosteroid therapy or plasmapheresis.

To illustrate the registry's operational structure, screenshots of key interface components—such as the patient summary page, treatment timeline display, and clinical data entry forms—are presented in the Supplementary material.

For the present analysis, a dataset comprising all records from February 1, 2013, to January 1, 2024, was extracted on June 17, 2024. The data were processed and analyzed using Microsoft Access and IBM SPSS Statistics software. To compare non-parametric data, the Mann–Whitney *U* test and the chi-square test were used.

For comparative purposes, additional epidemiological and administrative data were obtained from the Hygiene Institute and the National Health Insurance Fund under the Ministry of Health of the Republic of Lithuania.

## Results

### Patient enrolment

**Figure 1:**
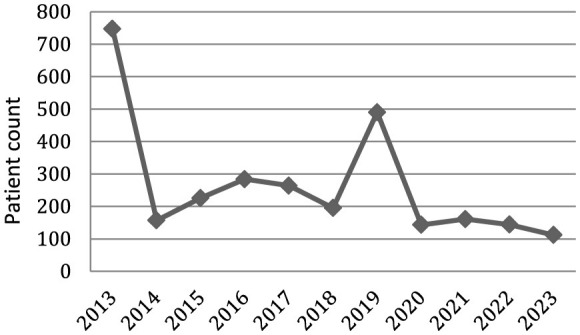
Annual enrollment of the Lithuanian MS Registry.

Annual patient enrollment varied markedly between 2013 and 2023 (Figure 1). Enrollment was highest in 2013 (747 patients), followed by a sharp decline in 2014 (157 patients). From 2015 to 2017, enrollment increased gradually, ranging from 226 to 284 patients, before decreasing again in 2018 (195 patients). A pronounced increase was observed in 2019 (490 patients), after which enrollment declined substantially in 2020 (143 patients) and remained relatively low and stable from 2021 to 2023 (112–161 patients per year).

At present, the registry includes 2,923 patients. According to data from the Hygiene Institute, 3,493 patients with MS were registered in Lithuania in 2023, corresponding to an estimated MS prevalence of 121.6 per 100,000 population.

Oligoclonal band (OB) testing in cerebrospinal fluid was performed in 1,263 patients, with OB detected in 1,043 cases (82.6%).

### Follow-up activity and data completeness

Follow-up activity was used as an indicator of data recency. Patients were classified as having active follow-up if at least one visit had been recorded since January 1, 2021, and as having inactive follow-up otherwise. Detailed characteristics of these groups are presented in Table 1.

**Table 1:** Characteristics of “active follow-up” multiple sclerosis patients in the Lithuanian MS Registry compared to patients without recorded visits since 2021.

**Characteristic**	**Active follow-up**	**Inactive follow-up**	***p-*value**
All	1,651	1,272	
Women, *n* (%)	1,082 (65.5)	852 (67.0)	0.082
Median of age, (min-max)^*^, years	46 (19–80)	56 (23–91)	< 0.001
Median of age at first visit (min-max), years	34 (10–75)	38 (12–77)	< 0.001
Duration of follow up^**^, median (min-max), years	5 (−2 to 11)	0 (−20 to 7)	< 0.001
Duration from first visit to registry inclusion median (min-max), months	24 (−2 to 481)	74.5 (0–392)	< 0.001
Number of patient visits, median (min-max)	21 (1–122)	10 (1–95)	< 0.001
Median frequency of visits per year (min-max)	3 (0.4–10.2)	0.9 (0.04–5.3)	< 0.001
MS clinical courses			
RR, *n* (%)	922^a^ (55.8)	238^b^ (18.7)	< 0.001
PP, *n* (%)	40^a^ (2.4)	42^a^ (3.3)	
SP, *n* (%)	55^a^ (3.3)	116^b^ (9.1)	
Unknown, *n* (%)	634^a^ (38.4)	876^b^ (68.9)	
EDSS during first visit, median (min.-max)	2.5 (0–8.5)	3.0 (0–8.5)	< 0.001
EDSS during last visit, median (min-max)	3.0 (1–8.5)	3.5 (0–7.5)	< 0.001
Use of disease modifying treatment, *n* (%)	1394^a^ (84.4)	373^b^ (29.3)	< 0.001
Treatment with AHSCT, *n* (%)	17^a^ (1.0)	9^a^ (0.7)	
Patients who have at least one registered relapse, *n* (%)	1,536 (93.0)	999 (78.5)	0.006
Number of relapses per person, median (min-max)	3 (1–25)	3 (1–25)	
Treated with corticosteroids, median (min-max)	2 (1–19)	2 (1–22)	
Treated with plasmapheresis, median (min-max)	1 (1–10)	2 (1–23)	

RR, relapsing-remitting; PP, primary progressive; SP, secondary-progressive; EDSS, Expanded Disability Status Scale; AHSCT, autologous hematopoietic stem cell transplantation. ^*^Age at the time the data was received. ^**^Calculated based on the date of the last visit and the date on which the patient was registered in the registry. a,b Superscript letters denote groups that differ significantly in post-hoc chi-square comparisons.

A total of 1,651 patients (47.3%) had active follow-up. Patients in the active follow-up group were younger (median age 46 vs. 56 years; *p* < 0.001) and had a younger age at first visit (median 34 vs. 38 years; *p* < 0.001). The sex distribution did not differ significantly between groups (*p* = 0.082).

The duration from first visit to registry inclusion was shorter among patients with active follow-up (median 24 vs. 74.5 months; *p* < 0.001). These patients also had a longer duration of follow-up (median 5 vs. 0 years; *p* < 0.001), a higher number of recorded visits (median 21 vs. 10; *p* < 0.001), and a higher median visit frequency per year (3.0 vs. 0.9; *p* < 0.001).

The distribution of MS clinical courses differed significantly between the groups (*p* < 0.001). The proportion of patients with an unknown clinical course was higher in the inactive follow-up group (68.9 vs. 38.4%).

Markers of disability also differed between groups. Patients with active follow-up had lower EDSS scores at both the first (median 2.5 vs. 3.0; *p* < 0.001) and last recorded visit (median 3.0 vs. 3.5; *p* < 0.001). Moreover, within the active follow-up group, 88.8% of patients had a last recorded EDSS score below 6, indicating that the vast majority remained in the mild-to-moderate disability range.

**Figure 2:**
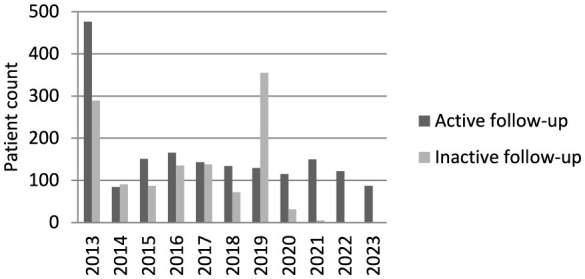
Annual enrollment by follow-up status (active and inactive follow-up groups).

The annual numbers of registered patients with active and inactive follow-up are shown in Figure 2.

Overall, nine deaths were recorded in the MS Registry. According to data from the Hygiene Institute, 25 deaths among patients with MS were reported in Lithuania in 2023.

### Disease-modifying treatment

Use of disease-modifying therapy (DMT) was more common among active follow-up patients (84.4 vs. 29.3%; *p* < 0.001). As shown in Table 2, 1,394 active follow-up patients were receiving DMTs. Platform therapies—including first-line injectables, teriflunomide, and dimethyl fumarate—accounted for more than half of all treatments. Among biologic therapies, ocrelizumab was the most frequently used (14.3%), followed by ofatumumab (5.4%), natalizumab (3.2%), and alemtuzumab (1.9%). In the active follow-up group, the median duration of treatment with the same DMT was 3 years, with the longest continuous treatment observed for interferon beta-1a (23 years). Comparison with national reimbursement data for 2023–2024 demonstrated similar treatment distributions. Overall, the registry captures approximately 69.5%−74.6% of all patients receiving DMT in Lithuania.

**Table 2:** Distribution of patients receiving disease-modifying therapy.

**DMT**	**“Active follow-up” patients who received DMT, *n* (%)**	MS patients who received DMT in 2023^*^, *n* (%)	MS patients who received DMT in 2024^*^, *n* (%)
First-line injectable DMTs	366 (26.3)	444 (23.8)	416 (20.7)
Teriflunomide	184 (13.2)	305 (16.3)	306 (15.2)
Dimethyl fumarate	188 (13.5)	309 (16.5)	279 (13.9)
Fingolimod	76 (5.5)	123 (6.6)	106 (5.3)
Ponesimod	29 (2.1)	47 (2.5)	109 (5.4)
Siponimod	30 (2.2)	33 (1.8)	23 (1.1)
Cladribine	160 (11.5)	131 (7.0)	122 (6.1)
Ocrelizumab	199 (14.3)	373 (20.0)	394 (19.6)
Ofatumumab	75 (5.4)	59 (3.2)	212 (10.6)
Natalizumab	60 (4.3)	44 (2.4)	40 (2.0)
Alemtuzumab	27 (1.9)	–	–
Over all	1,394	1,868	2,007

MS, multiple sclerosis; DMT, disease-modifying therapy.

^*^Data from the National Health Insurance Fund under the Ministry of Health.

## Discussion

This report describes the rationale for establishing the Lithuanian Multiple Sclerosis Registry and summarizes the scope of data collected to date. Although the registry has been in place for over a decade, it continues to face challenges in achieving representative patient coverage and ensuring high data quality. These limitations are partly attributable to the lack of integration with existing clinical documentation systems and the absence of systematic quality control procedures.

Analysis of enrollment patterns indicates marked variation over time. The initial peak in enrollment corresponds to the early phase of registry activity and likely reflects the inclusion of large volumes of retrospectively collected data. A second peak observed in 2019 may be attributable to intensified data entry during the COVID-19 pandemic, when reduced routine clinical activity during lockdown periods may have allowed clinicians more time to compile and submit registry data. In more recent years, patient enrollment has shown a declining trend.

In Lithuania, DMTs are prescribed exclusively at specialized MS centers. Patients who are not treated with DMTs are typically managed in primary care, which increases the likelihood that patients receiving DMTs are captured in the registry. This differential inclusion suggests the presence of selection bias and may limit the generalizability of registry-based analyses. Although the registry includes approximately 69.5%−74.6% of patients receiving DMTs nationwide, only 47.3% of the estimated MS population in Lithuania has active follow-up recorded, indicating incomplete longitudinal coverage. Notably, despite reimbursement restrictions, nearly one third (32.2%) of all MS patients receiving DMTs in Lithuania are treated with biological therapies.

Data completeness represents another important limitation. A substantial proportion of cases lack information on key clinical variables, particularly disease course, with missing data more frequent among patients with inactive follow-up. Discrepancies in mortality reporting further illustrate challenges in data completeness, as the number of deaths recorded in the registry is considerably lower than figures reported by national statistical sources. Together, these findings emphasize the need for improved mechanisms for systematic data updating and validation.

The registry is also constrained by the limited range of collected variables. Notably, the absence of brain MRI data restricts its usefulness for detailed clinical characterization and advanced research applications, particularly in studies of disease progression and treatment response.

Furthermore, increased efforts and closer cooperation are needed to develop the database infrastructure and software and distribute the tool across all participating centers. Providing comprehensive training and ongoing support will be essential to encourage widespread adoption and ensure proper use. Additionally, continuous maintenance, regular updates, and systematic monitoring must be implemented to guarantee data quality, security, and effective utilization over time. Sustained collaboration and clear communication between technical teams, physicians, and administrators will maximize the tool's potential and ultimately improve patient care and research outcomes.

Successful examples from other countries can serve as motivation for improvement. The Czech National Registry (ReMuS) began its activities in 2013 and has since demonstrated consistent growth in patient enrollment, reaching 17,478 actively monitored individuals out of an estimated 22,000 MS patients in the Czech Republic by 2021—approximately 79.4% (15). In comparison, our registry—although initiated around the same time—continues to face challenges in patient recruitment, currently covering approximately 55% of all registered MS patients in Lithuania, and 47.3% of patients in active follow-up. The Swedish MS Registry has contributed data to over 100 scientific reports and has recently published a validation of the registry (16, 17).

Patient-reported outcome platforms used in the UK and Finnish registries further enhance data collection for both clinical care and research, while the Norwegian registry's integration of a biobank supports translational research efforts (18–22).

International initiatives also provide guidance for future improvements. An expert workshop organized by the U.S. National Institute of Neurological Disorders and Stroke and the National Multiple Sclerosis Society produced strategic recommendations for optimizing the use of MS cohort data, particularly in progressive MS research (23). The recently published MS Data Alliance Core Dataset guides offer practical tools for restructuring dataset categories and variables to align with international standards (24). Adoption of these recommendations could enhance data comparability, facilitate international collaboration, and enable participation in broader research networks and data-sharing initiatives.

## Conclusions

The Lithuanian MS Registry captures a substantial proportion of the national MS population and provides valuable longitudinal monitoring data. Notably, Lithuania is the only country among the three Baltic states to have a dedicated MS Registry, representing an important first step in structured MS data collection and monitoring. The registry faces challenges related to the comprehensive enrollment of all MS patients across the country, as well as issues concerning data completeness and accuracy. A decline in patient recruitment and inconsistencies with external data sources underscore the need for improved data integration and robust quality assurance measures. Strengthening validation processes is essential to ensure the registry's reliability and utility in both clinical practice and research settings.

## Data Availability

The datasets presented in this article are not readily available because parts of this material are based on data and/or information compiled and provided by the Hygiene Institute and the National Health Insurance Fund under the Ministry of Health of the Republic of Lithuania, which are publicly available. The dataset from the Lithuanian MS Registry is held securely in coded form and is not accessible to the public. Requests to access the datasets should be directed to dalius.jatuzis@santa.
