# Supplementary material for: The Lithuanian multiple sclerosis registry: current framework and quality challenges
Source: Front Neurol. 2026 Feb 23;17:1728596. doi: 10.3389/fneur.2026.1728596 (PMC12967925; doi:10.3389/fneur.2026.1728596)
Supplement: Supplementary file 1 [file Data_Sheet_1.docx]

**Supplementary Material**

A

B

A-Patient registration form

B-Patient record view
